# The forgotten tale of immunoglobulin allotypes in cancer risk and treatment

**DOI:** 10.1186/2162-3619-2-6

**Published:** 2013-02-20

**Authors:** Janardan P Pandey, Zihai Li

**Affiliations:** 1 Department of Microbiology and Immunology, Medical University of South Carolina, Charleston, SC, USA; 2 Hollings Cancer Center, Medical University of South Carolina, Charleston, SC 29466, USA

**Keywords:** GM and KM allotypes, IGHG genes, ADCC, CDC, Isoallotypes, Immunosurveillance, GVL

## Abstract

Monoclonal antibody (mAb) has fulfilled the promise of being the “Magic Bullet” in oncology with the clinical success of mAbs against CD20, Her-2/neu, epidermal growth factor receptor, vascular endothelial cell growth factor and others in a variety of cancers. Most manufacturers of mouse-human chimeric antibodies (and most immunologists) have treated the constant region of human immunoglobulin (Ig) as if it were naturally monomorphic and therefore not immunogenic in humans. In fact, the constant region of Ig heavy and light chain is highly polymorphic, and yet Ig haplotypes are usually not defined by genome-wide association studies nor are they considered to be important for optimizing mAb therapy. We hereby summarize evidence that Ig allotypes are important and biologically relevant in that they contribute to the etiopathogenesis of many malignant, infectious, and autoimmune diseases. Because Ig allotypes differ from each other in engaging Fc receptor, we argue that future development of effective mAb therapy for cancer should take a patient-specific approach by using the correct allotype for each patient to maximize the efficacy of this therapy.

## Introduction

**Figure 1:**
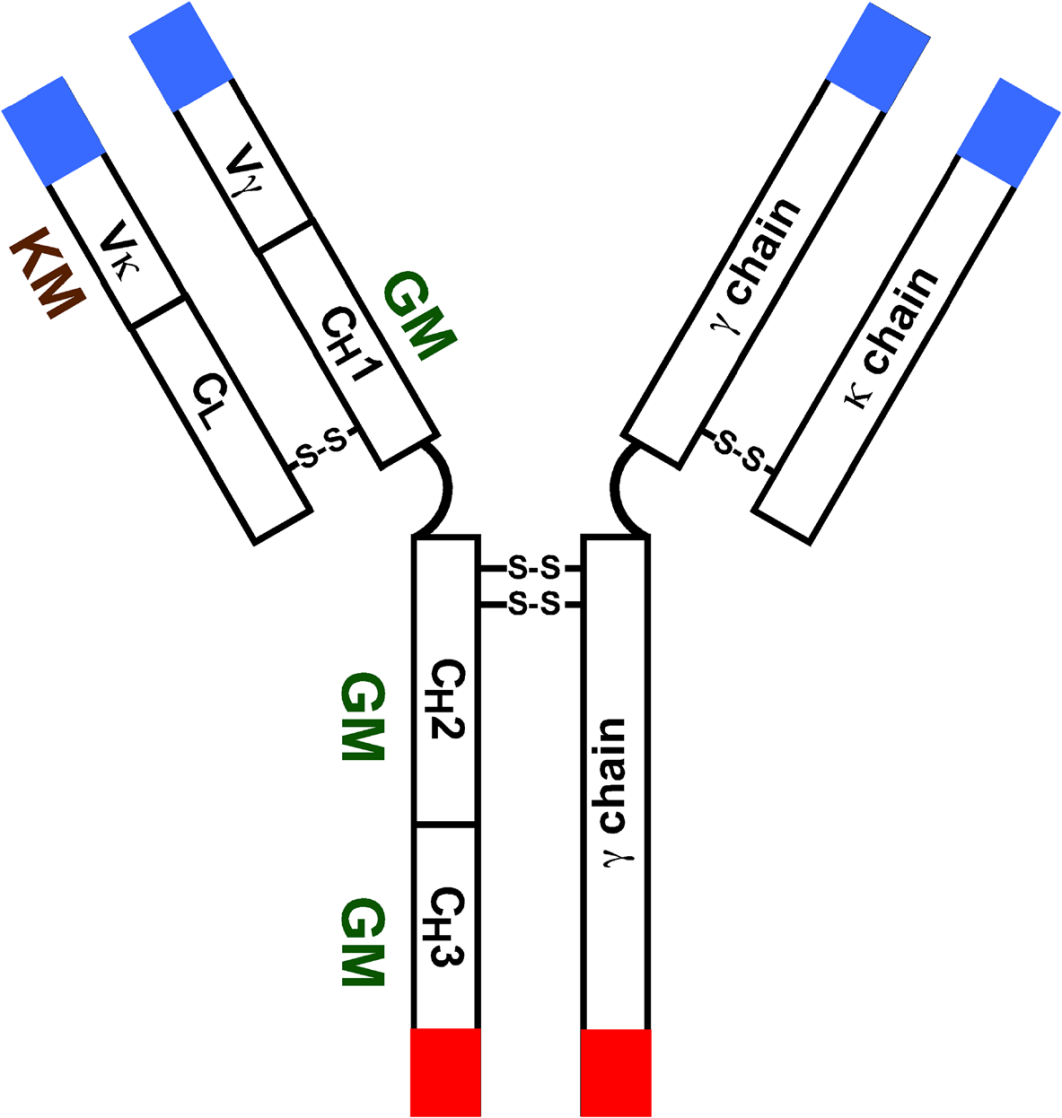
Localization of GM and KM allotypes on IgGκ molecule.

Though any genetic variant of a protein could be called an allotype, in immunology, the term is commonly used for hereditary antigenic determinants expressed on immunoglobulin (Ig) polypeptide chains. Allotypes are encoded by autosomal codominant genes that follow Mendelian laws of heredity. With one exception [1], allotypes identified thus far are expressed on the constant (C) region of IgG, IgA, and IgE heavy chains and on κ-type light chains [2]. In this minireview, we will focus primarily on GM (genetic markers of γ chain) and KM (genetic markers of κ chain) allotypes (Figure 1).

## GM allotypes

GM allotypes are encoded by three very closely linked and highly homologous genes—Ig heavy chain G1 (*IGHG1*), *IGHG2*, and *IGHG3*—on chromosome 14q32. There are two systems of GM gene nomenclature currently in use—alphameric and numeric. We have provided both. In accordance with the international system for human gene nomenclature, haplotypes and genotypes/phenotypes are written by grouping together the markers that belong to each subclass, by the numerical order of the marker and of the subclass; markers belonging to different subclasses are separated by a space, while allotypes within a subclass are separated by commas. There are currently 18 serologically testable GM specificities—four on γ1 (1/a, 2/x, 3/f, 17/z), one on γ2 (23/n), and 13 on γ3 (5/b1, 6/c3, 10/b5, 11/b0, 13/b3, 14/b4, 15/s, 16/t, 21/g1, 24/c5, 26/u, 27/v, 28/g5). With the exception of allelic GM3 and GM17 determinants expressed in the Fd region (the Fab portion of heavy chain), all other GM alleles are expressed in the Fc region of γ chains. Linkage disequilibrium (non-allelic association) in the GM system within a racial group is almost absolute and the determinants are transmitted as a group—haplotypes. Each major race has a distinct array of several GM haplotypes. GM 3 23 5,10,11,13,14,26 and GM 1,17 5,10,11,13,14,17,26 are examples of common Caucasian and Negroid haplotypes, respectively. Unless there is genetic admixture, these two groups do not share any haplotypes. Nucleotide substitutions (SNPs) responsible for most of the 18 serologically detectable GM specificities have not yet been identified. Serological reagents for GM typing are either extremely scarce or not available at all. Molecular methods for determining some GM markers are available; we, and others, are currently developing DNA-based methods for other markers.

## Isoallotypes

These are markers that behave as alleles in one IgG subclass (allotypes) but are also expressed in all molecules of at least one other subclass (isotype). For instance, human γ4 chains, unlike other γ chains, do not express unique allotypes, but they do express isoallotypes. These isoallotypes may be functionally involved in the so-called “Fab arms exchange” between IgG4 antibodies, an immunological mechanism implicated in the anti-inflammatory activity of these antibodies [3]. The arginine/lysine substitution at amino acid position 409 (R409/K409) of γ4, characterizes an isoallotype [4]. R409 and K409 behave as alleles on γ4 (allotypes), but they are also present on all molecules of the other γ chains (isotypes). R409 enables the Fab arms exchange, while K409 blocks it [5]. Thus, examination of γ allotypes and isoallotypes may shed light on the etiology of IgG4-mediated diseases [6].

## GM allotypes and disease susceptibility

The marked differences in the frequencies of GM allotypes among races, strong linkage disequilibrium within a race, and racially-restricted occurrence of GM haplotypes, all suggest that differential selection over many generations may have played an important role in the maintenance of polymorphism at these loci. As first suggested by J.B.S. Haldane, major infectious diseases have been the principal selective forces of natural selection [7]. Malignant diseases, however, might also have exerted adaptive pressure on GM polymorphisms. Since most cancers (breast, prostate, etc.) occur predominantly in middle and older age groups, it is commonly suggested that they may not be subject to natural selection because people with these diseases are beyond their reproductive age. However, the predominant occurrence of cancer in the older age groups may reflect the multistep nature of cancer development rather than the lack of evolutionary adaptive pressure [8].

Using hypothesis driven candidate gene approaches, numerous studies have identified particular GM genes as risk factors for many malignant [9-13], infectious [14-18], and autoimmune diseases [19-24], but most of these findings have not been confirmed *or refuted* by modern genome-wide association studies (GWAS). One contributing factor might be the absence of GM gene probes in most genotyping platforms. GWAS are assumed to be able to detect/tag all SNPs in the genome whose frequency is at least 5% or less (using newer arrays). This, however, is not true. Most GM alleles are common within a racial group (some with gene frequency >70%), but the *IGHG* gene segments harboring them are highly homologous and apparently not amenable to the high throughput genotyping technology used in GWAS. Because these genes were not typed in the HapMap project, they cannot be imputed or tagged (through linkage disequilibrium) by any SNPs that are included in the genotyping platforms. One of us (J.P.P.) has stressed the importance of GM genes in human biology and pointed out that they are not being evaluated by GWAS in letters to some high-profile journals [25-28], hoping to reach a wide audience. It is encouraging to note that a recent GWAS of multiple sclerosis did include GM alleles and concluded that particular GM haplotypes contributed to the higher IgG levels in the cerebrospinal fluid of these patients [29]. Using a candidate gene approach, we came to the same conclusion over three decades ago [30].

## Possible mechanisms underlying the involvement of GM genes in the etiopathogenesis of human diseases

Several immunological mechanisms, which are not mutually exclusive, can be postulated to explain GM gene involvement in various human diseases.

## GM allotypes and immune response to self and non-self antigens

GM allotypes could mediate the development or progression of a disease by influencing the immune responsiveness to the antigens relevant to the disease. Importance of Ig allotypes in controlling immune responsiveness was recognized over 40 years ago [31]. More recent studies have shown that immune responsiveness to a variety of antigens—infectious agents, vaccines, autoantigens, including some tumor-associated antigens, are associated with particular GM and KM (see below) allotypes [14,32-39]. We have recently reported the contribution of these genes to antibody responses to the tumor-associated antigens mucin 1 and human epidermal growth factor receptor 2 (HER2) [40-43].

GM markers could influence antibody responsiveness to disease-associated antigens by being part of the recognition structure for these antigens on the B-cell membrane-bound IgG. Membrane-bound IgG molecules expressing different GM specificities may have differential affinity to antigenic epitopes, resulting in stronger/weaker humoral immunity to particular antigens. Alternatively, these C-region determinants could influence the conformation of the Ig variable (V) regions involved in antigen binding and thus cause changes in antibody affinity and specificity. Studies in mice investigating the contribution of C-region determinants to the expression of certain idiotypes and their participation in other conformational changes in the V region support this interpretation. Involvement of both C and V regions in the formation of idiotypic determinants was documented many years ago [44]. Recent investigations by Casadevall and his colleagues have clearly established that the C region contributes to the affinity and specificity of antibodies [45]. Relevant to the present discussion, they have shown that amino acid sequence polymorphisms in the C region of the Ig molecule affect the secondary structure of the antigen-binding site in the V region [46]. Amino acid substitutions associated with GM allotypes cause structural changes in the C region, which could impose structural constraints (conformation) on the V region, resulting in variation in antibody affinity and specificity. Thus, C regions expressing different GM allotypes, even when combined with identical V region sequences, can generate new antibody molecules with new functions.

## GM allotypes and antibody-dependent cell-mediated cytotoxicity (ADCC)

ADCC, which links the innate and the adaptive arms of immunity, is a major host immunosurveillance mechanism against tumors, as well as the leading mechanism underlying the clinical efficacy of therapeutic antibodies such as cetuximab and trastuzumab, which target tumor antigens, HER1 and HER2, respectively. IgG antibody mediated ADCC is triggered upon ligation of Fcγ receptor (FcγR) to the Fc region of IgG molecules. It follows that genetic variation in FcγR *and* Fc could contribute to the differences in the magnitude of ADCC. Several studies have shown that genetic variation in FcγR contributes to the differences in the magnitude of ADCC [47-50], but with the exception of our studies, the contribution of natural genetic variation in the Fc region of IgG—GM allotypes—has not been investigated. Using an ADCC inhibition assay, we have shown that IgG1 expressing the GM 3+,1-,2- allotypes was equally effective in inhibiting cetuximab- and trastuzumab-mediated ADCC of respective target cells, in the presence of NK cells expressing either valine or phenylalanine allele of FcγRIIIa [51]. These findings have important implications for engineering antibodies with human γ1 C region. Concerted effort is currently being directed at engineering Fc variants with optimized affinity for activating and inhibiting FcγRs [52-54]. Evaluation of the role of naturally occurring Fc (GM) variants that may have been evolutionarily selected because of their contribution (through ADCC and other protective immunosurveillance mechanisms) to survival from malignant diseases [8] is essential for engineering the next generation of humanized monoclonal antibodies, which have reduced immunogenicity, have better clinical efficacy, and benefit more patients than what is possible with the currently available therapeutics.

## GM allotypes and complement-dependent cytotoxicity (CDC)

The complement system plays an important role in immunosurveillance, and CDC has been shown to be instrumental in the efficacy of certain mAbs, such as rituximab (anti-CD20) and alemtuzumab (anti-CD52). Though not yet investigated, there is a good rationale for the involvement of GM alleles in CDC as well. In CDC, C1q binds the antibody and triggers the complement cascade. C1q’s binding affinity to the antibody molecules is likely to affect the level of CDC against tumor cells. It has been known for some time that C1q discriminates between two major alleles of IgG3: It binds slightly better to IgG3 proteins expressing the GM21 allele than to those expressing the alternative GM5 allele [55]. It follows that IgG3 mAbs expressing the GM21 allele in their Fc would be more effective in CDC against cancer cells.

## GM allotypes and viral immunoevasion

Several viruses have been implicated in the etiopathogenesis of malignant diseases, and the list of virally-induced/spurred cancers is growing steadily. To ensure their survival, viruses must be able to enfeeble the defense mechanisms employed by the host’s immune system to eliminate the virions and virally infected cells (immunosurveillance). During the co-evolution of viruses and their hosts, the host must have evolved specific mechanisms to modulate the effects of these viral strategies and ensure our survival as a species. A clue to one such mechanism is offered by studies involving GM allotypes and human cytomegalovirus (HCMV), which is implicated in gliomas [56], and hepatitis C virus (HCV), a well-known etiological agent for liver cancer.

HCMV has evolved a large repertoire of immune evasion strategies. One strategy involves generating two proteins—encoded by genes *TRL11/IRL11* and *UL119-UL118*—that have functional properties of the FcγR [57], which may enable the virus to evade host immunosurveillance by evading the effector consequences of antibody binding, such as ADCC, CDC, and phagocytosis. We have recently shown that GM alleles modulate this viral strategy: the HCMV *TRL11/IRL11*-encoded FcγR has significantly higher affinity for IgG1 proteins expressing the GM 3+,1-,2- allotypes than for those expressing the allelic GM 17+,1+,2+ allotypes [58]. Because of their higher affinity to the HCMV-encoded FcγR, anti-HCMV IgG1 antibodies expressing the GM 3+,1-,2- allotypes would be more likely to have their Fc domains scavenged, thereby reducing their immunological competence to eliminate the virus through Fc-mediated effector mechanisms. Consequently, the frequency of these allotypes would be expected to be higher in patients with HCMV-induced/spurred diseases. This appears to be the case in glioma (manuscript under review). Another herpes virus, herpes simplex virus type 1 (HSV1), also encodes for immune-evading FcγR proteins that discriminate between GM alleles [59]. However, the HCMV- and HSV1-encoded FcγRs have contrasting binding affinities to allelically disparate IgG1 antibodies, making the particular alleles relevant to the etiology of HCMV- or HSV1-spurred diseases. Similarly, the HCV core protein, which also displays the functional properties of the FcγR, binds differentially to IgG proteins expressing different allotypes [60-62], making these allotypes relevant to the etiology of HCV-induced liver cancer.

## Immunoglobulin KM allotypes

Like the γ chains, the κ chain is also polymorphic, characterized by the segregation of three alleles—KM1, KM1,2, and KM3 on chromosome 2p12 [2]. Over 98% of the people positive for the KM1 allotype are also positive for KM2; the KM1 allele, without KM2, is extremely rare. These alleles represent amino acid substitutions at positions 153 and 191 of κ chain—KM1: valine 153, leucine 191; KM1,2: alanine 153, leucine 191; and KM3: alanine 153, valine 191.

Though virtually ignored so far, KM alleles are likely to become important in cancer immunology research, thanks to a major genomics study [63]. This comprehensive analysis of human gene expression identified *IGKC* as a novel prognostic marker in several solid tumors. The *IGKC* as a single marker had as much effect on metastasis-free survival as the 60 genes in the B-cell plasma cell metagene. This is not surprising since the κ chain can pair with the Ig heavy chains of all classes and subclasses. Although the authors could not address the biological roles of the *IGKC* signature, their results provide a compelling rationale for investigating the role of KM alleles, genetic variants of *IGKC*, in humoral immunity to tumor-associated antigens. It is relevant to note that several years ago we noted an increased frequency of the KM1 allele in patients with head and neck cancer [64]. Examination of KM alleles would be especially important in malignancies characterized by racial disparity, such as prostate cancer, since KM gene frequencies differ significantly among various racial groups [2].

It would also be important to investigate possible interactive effects of GM and KM alleles in disease susceptibility, in immune responsiveness to tumor antigens, and in patient survival after therapy. Although immunology textbooks state that heavy and light chains pair randomly to produce Ig molecules, some studies in experimental animals have provided evidence for preferential pairing of these polypeptide chains [65,66]. Thus γ and κ chains expressing particular GM and KM alleles could preferentially associate to generate an IgG antibody directed against an antigen. We have shown such interactive effects of GM and KM alleles in humoral immunity to Epstein-Barr virus [67], group B streptococcus antigens [68], HCV envelope proteins E1 and E2 [32], and mucin 1 [40].

## KM allotypes and graft versus leukemia (GVL)

KM allotypes appear to be relevant to hematological malignancies. Since they are expressed on B cells, they are potential minor histocompatibility antigens and could be targets for the GVL phenomenon when hematopoietic cell transplant (HCT) recipients and donors express different KM alleles. Results of a study from Australia support this contention [69]. In this study, HLA-matched Caucasian donors and recipients of HCT for B-cell malignancies were typed for KM alleles to determine whether or not KM disparity influenced the HCT outcome. KM allotype disparity between transplant pairs was associated with increased survival compared with pairs that were not mismatched. More such studies are needed, especially in African Americans where KM1 allele frequency is significantly higher than that in Caucasians.

## GM and KM allotypes and development of resistance to monoclonal antibody therapy

The response rate to most mAbs is low and all patients eventually develop resistance to this therapy. Numerous mechanisms of resistance have been proposed but they do not account for the total inter-individual variation in treatment responses in *de novo* and in acquired resistance, which suggests involvement of additional mechanisms. One potential mechanism that has not received adequate attention is the role of anti-allotype antibodies. All licensed chimeric or humanized mAbs express certain GM allotypes on their heavy chains and KM allotypes on their κ light chains. For instance, trastuzumab expresses GM17 and KM3 and cetuximab expresses GM3 and KM3. Most GM/KM determinants are highly immunogenic, and the Ig molecules carrying these markers cross the maternal-fetal barrier in both directions, leading to anti-GM/KM antibody production in the mother against the paternal GM/KM markers present in the child, and in the child against the maternal GM/KM alleles [70]. Patients who lack the GM/KM allotypes present on the mAbs would be expected to generate antibodies to these determinants if exposed through maternal-fetal incompatibility, allotype-incompatible blood transfusion or infusion of the mAbs. These preexisting or mAbs-induced anti-allotype antibodies and the administered mAbs could form immune complexes that would be eliminated by phagocytic cells, leading to nonresponsiveness. At present, no data are available on the prevalence of anti-allotype antibodies in patients treated with mAbs.

In summary, inclusion of polymorphic GM, KM, and FcγR alleles in cancer immunology investigations could identify novel immune pathways to tumor immunity. This knowledge would be helpful in diagnosis, prognosis, and in devising effective immunotherapeutic strategies against cancer. At present, a candidate gene approach would be necessary for these studies, since these genes (GM in particular), are not included in most genotyping arrays used in GWAS. Furthermore, GWAS, in general, do not measure epistasis (gene-gene interaction), which probably accounts for a significant portion of the “missing” heritability in complex diseases.

## Competing interests

The authors declare that they have no competing interests.

## Authors’ contributions

JPP and ZL wrote the manuscript. Both authors read and approved the final manuscript.
